# Factors Influencing Individual Variation in Farm Animal Cognition and How to Account for These Statistically

**DOI:** 10.3389/fvets.2018.00193

**Published:** 2018-08-17

**Authors:** Emily V. Bushby, Mary Friel, Conor Goold, Helen Gray, Lauren Smith, Lisa M. Collins

**Affiliations:** Faculty of Biological Sciences, University of Leeds, Leeds, United Kingdom

**Keywords:** cognition, refinement, individual, welfare, livestock, multilevel modeling

## Abstract

For farmed species, good health and welfare is a win-win situation: both the animals and producers can benefit. In recent years, animal welfare scientists have embraced cognitive sciences to rise to the challenge of determining an animal's internal state in order to better understand its welfare needs and by extension, the needs of larger groups of animals. A wide range of cognitive tests have been developed that can be applied in farmed species to assess a range of cognitive traits. However, this has also presented challenges. Whilst it may be expected to see cognitive variation at the species level, differences in cognitive ability between and within individuals of the same species have frequently been noted but left largely unexplained. Not accounting for individual variation may result in misleading conclusions when the results are applied both at an individual level and at higher levels of scale. This has implications both for our fundamental understanding of an individual's welfare needs, but also more broadly for experimental design and the justification for sample sizes in studies using animals. We urgently need to address this issue. In this review, we will consider the latest developments on the causes of individual variation in cognitive outcomes, such as the choice of cognitive test, sex, breed, age, early life environment, rearing conditions, personality, diet, and the animal's microbiome. We discuss the impact of each of these factors specifically in relation to recent work in farmed species, and explore the future directions for cognitive research in this field, particularly in relation to experimental design and analytical techniques that allow individual variation to be accounted for appropriately.

## Introduction

Understanding the cognitive capabilities of animals, how they may be affected by the environments in which we keep them, and the extent to which these changes can be used as an indicator of welfare, are increasingly of interest in the field of animal welfare. Cognition has been broadly defined as any process or mental action required to gain, process, and use information collected via experience, thought and senses (1). This includes functions such as attention, memory, social learning, associative learning, judgment, and reasoning, to name a few (2). Understanding the cognitive capabilities of animals thus allows us a window into the way in which an animal perceives and makes sense of the world around it. This is particularly important for farmed species, which there are large numbers of globally, and where the husbandry practices directly affect their welfare and have the potential to cause suffering (3, 4). There are numerous tests available that allow us to assess cognitive ability (Table 1) and multiple factors that we know are important to consider in applying these. Despite advances in this endeavor, assessing and interpreting cognition in non-human animals is challenging.

**Table 1:** An overview of cognitive tests that have previously been used in farmed species and the type of cognitive ability they assess.

**Cognitive ability**	**Task**	**References**
Spatial cognition	Learning distribution/position of baited locations	*Sheep:* (5, 6); *Cattle*: (7, 8); *Chickens*: (9–11); *Fish:* (12)
	Parallel arm maze	*Cattle*: (13); *Fish:* (14)
	Radial arm maze	*Pigs:* (15, 16); *Chickens*: (17, 18)
	Spatial maze with zones	*Sheep:* (19–21); *Pigs:* (22) *Cattle*: (23–25); *Fish*: (26, 27)
	T-maze	*Cattle:* (28); *Chickens*: (17, 29); *Fish:* (30, 31)
	Y-maze	*Chicken*: (17); *Sheep*: (32); *Cattle:* (33)
	Rotating enclosure	*Chickens:* (34, 35)
Memory	Holeboard spatial discrimination	*Pigs:* (36–39); *Chickens:* (29)
	Object recognition	*Pigs:* (40)
	Delayed match to sample	*Chickens:* (41)
	Devaluation foraging technique	*Chickens:* (42, 43);
	Delayed search task	*Chickens:* (44, 45)
	Two step foraging task	*Goats:* (46)
Social cognition	Foraging arena task	*Pigs:* (47, 48)
	Follow knowledgeable individual	*Pigs:* (49)
	Mirror task	*Pigs:* (50, 51) *Sheep:* (52)
	Y-maze	*Pigs:* (53–55); *Sheep*: (56, 57); *Chickens:* (58, 59); *Cattle:* (60)
	Social recognition test	*Pigs:* (61)
	Social recognition based on visual/olfactory cues–operant tasks	*Chickens*: (62); *Cattle:* (63, 64); *Sheep:* (65); *Chickens:* (59)
	Choice test	*Fish:* (66)
Social learning	Distance to aversive/gentle handler	*Cattle:* (67)
	Operant task	*Cattle:* (68)
	Food choice test	*Pigs:* (69); *Chickens:* (70)
	Object choice test	*Goats:* (71)
	T-maze	*Goats:* (72)
	Detour task	*Goats:* (73)
Inferential reasoning	Preferential looking paradigm choice test	*Goats:* (74)
	Object choice task	*Goats, pigs:* (75–77)
Discrimination learning	Image discrimination (visual discrimination)	*Pigs, Goats:* (55, 78, 79)
	Acoustic discrimination	*Pigs:* (80)
	Social discrimination (visual discrimination)	*Sheep:* (81)
Object permanence	Hidden reward object	*Pigs, Goats:* (82, 83)
	Perseveration error	*Goats:* (83)
Classical conditioning	Clicker training	*Cattle:* (84)
	Eye blink response conditioning	*Sheep:* (85, 86)
	Trained to approach feed source with audio cues	*Cattle:* (87)
	Trace classical conditioning	*Chickens:* (88)
	Classical conditioning using light to signal arrival of food.	*Fish:* (89)
	Delay conditioning regime	*Fish:* (90)
Operant conditioning	Trained to approach feed source with audio cues	*Cattle:* (87, 91)
	Social contact motivation task	*Cattle:* (92)
	Nose wheel feeding task	*Pigs:* (93)
	Trained to urinate in a specific location	*Cattle:* (94)
Numerical understanding	Free-choice tests	*Chickens:* (95)
	Identification of trained rank-order target locations among identical alternatives	*Chickens:* (96)

Part of the difficulty with studying cognition in farmed animal species is due to individual variation in cognitive abilities. The majority of animal cognition studies examine how individuals perform in cognitive tasks on average, using aggregated data (e.g., the mean number of trials correct, average latency to respond). However, individuals also show between- and within-individual variation in cognition across repeated measurements or across different experimental conditions (97). Variation in individuals' average performance may represent relatively consistent between-individual differences in cognitive styles, similar to animal personality (98). Variation in within-individual cognitive change (e.g., the rate of learning) may further indicate differences in cognitive flexibility, a posited mechanism of behavioral plasticity (97). Variation in residual within-individual change (i.e., the amount of variation around an individual's average performance) could be used as a measure of cognitive resilience in farmed animals [e.g., (99)]. In turn, factors such as age, sex, breed and personality may predict these facets of individual variation.

In this review, we explore the main causes of within- and between-individual variation in cognitive testing of livestock. By “causes,” we mean factors that would result in individual variation all else being equal [e.g., (100)]. In section one, we give a brief overview of the types of cognitive tests available, including a table which shows the type of test and which cognitive function it has been used to assess. In section two, we then identify some key areas that can contribute to variation, including; sex, breed, personality, life stage, diet, mood, motivation, and gut microbiome. Following this, in section three, we highlight statistical methods for estimating different facets of individual variation and include a statistical example.

## Types of cognitive tests

There are multiple ways to assess an animal's cognitive capabilities and in Table 1 we list some of the most common tests used to investigate different aspects of cognition in farmed animal species. However, many require the use of more than one cognitive ability and tests are often adapted to suit the species or cognitive function of interest (101). Indeed, the notion of embodied cognition (102) highlights the connection and interaction of the brain with the body and the environment. The anatomical features of a species may allow it to perform better or worse on a given cognitive task than another species. Thus, to accurately measure cognition with cognitive tasks, it is necessary to ensure that the task is designed to suit the physical abilities of the species being tested. It should also be noted that, when exploring sources of variation in cognitive testing, the test itself could be a contributing factor. One example of this is side preference, which can develop in T-maze or Y-maze study designs, as shown in a variety of species including sheep (103), rats (104), and cows (105). In these cases, the learning outcomes of the task can be overshadowed by the animal's preference to occupy one side of the maze. Furthermore, farm animals often have not had an extensive period of regular positive contacts with humans, as may be the case for companion animals. Therefore, in order to conduct cognitive testing in these animals, habituation to the test set-up will be required to ensure that the responses measured are task specific and not affected by fear of either the experimenters or the testing situation. Regardless of the cognitive task employed, determining which factors are causing individual variation in cognition requires distinguishing between direct causal relationships and correlations.

## Factors contributing to individual variation in cognitive performance

Cognition and behavior are hierarchically organized and continuously interacting with endogenous (e.g., life stage) and exogeneous (e.g., the behavior of conspecifics, developmental environments) factors. This likely results in correlations between many different cognitive and behavioral variables [referred to as the “crud factor” in human psychology; (106)], as well as their significant associations with endogenous and exogenous variables, but these do not necessarily reflect direct causal relationships. As such, we interpret the “causes” of individual variation below as factors that result in variation between individuals who are equal in all other respects. This interpretation of causality follows Pearl's work (100) on distinguishing causal from associative relationships by determining which correlations in multivariate data disappear when all variables are conditioned on each other, i.e., finding conditional independence relations in the data [see (107) for an accessible review]. We also acknowledge that individual variation can emerge simply from the cumulative effects of unsystematic events occurring in the environment and/or in how individuals process information, even in genetically identical individuals [e.g., (108)]. This means that individual variation is to be expected a priori even when no obvious cause exists.

### Development and early life

During prenatal and neonatal stages of growth, there is a period of rapid brain development, including cell birth, migration, dendritic outgrowth, programmed cell death, and synapse production. The brain is particularly vulnerable to perturbations during development (109) and both endogenous and exogenous factors occurring at this time have been shown to influence cognition in livestock animals (110, 103). Prenatal stress is well-known to have an effect on the developing brain and on programming of the hypothalamic-pituitary-adrenal (HPA) axis (111–113). These effects have consequences for stress reactivity, behavior, and cognition in offspring that can continue throughout life (114). For example, ewes exposed to stressful situations during late pregnancy produced lambs that showed increased levels of fear and a decreased ability to navigate a maze, suggesting decreased spatial and working memory (21). Similarly, domestic chicks that experienced hypoxic conditions for 24 h during embryonic development had poorer performance in a bead discrimination task designed to test memory (115).

A further factor relating to development and cognition is an animal's origin litter. For example, Hernandez et al. (116) found that lambs from twins were more likely to change side preference in a two-armed maze test in comparison to singleton born lambs. The environment and experiences that an individual is subjected to during their developmental period are also influential. Calves fed using an enriched feeding method (instead of standard bucket feeding) showed decreased reactivity to novelty and, although initially they took longer to locate a reward, performed better in the reversal stage of a T-maze task (28).

Age and timing of weaning can also have implications for cognition and behavior. In livestock species, weaning often also involves separation from the mother, littermates and mixing into large groups of unfamiliar conspecifics in a new environment (117, 118). Weaning at earlier or later ages than the industry standard has been shown to influence stress (119) and behavior (120–123). Early weaning and material deprivation can significantly affect the brain and consequently cognition. For example, piglets that were weaned early at 10 days of age had decreased gene expression in the hippocampus (124). In other species, maternal deprivation can increase cell death in the brains of young rats (125) and can reduce neurogenesis in mice (126). Overall, these different life-stage factors can all influence cognitive function within an individual.

### Sex

Of all the factors considered here that may influence cognition, sex is perhaps one of the most evolutionarily well-conserved (127). In cognitive testing of farmed species, there has not been the same drive to detect sex-related effects as there has been in clinical trials on laboratory animals, so observed differences between sexes are typically reported incidentally rather than explicitly investigated. For example, Erhard et al. (103) found that male sheep required fewer runs to learn and solve a reversal-learning task than females at 18 months old. Conversely, another study with a similar maze design found female sheep were quicker to learn and solve a reversal-learning task than males at 4 months of age (116). However, this finding was not present by 18 months and, as the authors suggest, may only have reflected differing maturation rates of male and female animals.

Although sex has rarely been explicitly tested in farm animal cognition studies, statistically significant differences have been identified between the sexes for many biological parameters in clinical trials. Of particular note for this review are the studies that have identified sex differences in stress-related psychiatric disorders, such as depression, generalized anxiety disorder, acute and chronic post-traumatic stress disorder, with a higher risk of development in females than in males (128–131). Stress-related disorders are linked closely with cognitive alterations, and differing levels of performance in learning and memory tests in particular. For example, exposure to an acute or repeated stressful event is associated with enhanced learning in a classical conditioning task in male rats, but impaired performance in females (132); though this is only the case in adult females with mature oestrous cycles (133). By contrast, the opposite effect has been found in spatial learning and memory tasks, where acute stress exposure impairs males' performance in a Y maze, but enhances female rats' performances regardless of their oestrous cycle (134). Similar effects have been shown in another memory test, the Morris water maze test (135, 136). Even without the stress exposure, there are clear male advantages in spatial working and reference memory in rats that transcend strain, age, environment, and testing protocol differences. However, mouse studies have found a different pattern—that females have an advantage in water maze tests, but males have a small advantage in radial maze tests (137). Of relevance to the cognitive bias testing paradigm, risk seeking behavior in humans tested using a computerized balloon analog risk task, showed clear sex differences. Following exposure to an acute stressor, risk avoidance increased in females but risk seeking increased in males (138).

One consideration with the measurement of sex differences is that it is typically included as a binary variable and used as a simple to measure, catch-all, umbrella indicator for what is in reality a host of non-discrete, underlying interacting complex systems. As suggested by Maney (139), sex should be viewed as a proxy for as-yet unknown factors that co-vary with it, such as hormonal differences, sex-linked genetics, or experience. Testing simply for the existence of a difference between the sexes may mask distributional differences in co-varying variables, resulting in false negative outcomes; as such, it may be more informative to consider the extent to which the sexes differ, rather than whether or not a significant difference exists.

### Breed

The genetic composition of different livestock breeds has the potential to influence temperament, behavior, and cognition. The differences in cognitive task performance between different breeds may be due to factors that are not directly attributable to cognitive abilities *per se*. For example, temperament differences between breeds may alter the likelihood of an individual engaging with the task and/or influence their opportunities to be exposed to the stimulus (98). A study by Nordquist et al. (29) compared the responses of chickens breed for low mortality and a control breed/line in multiple cognitive tests, including the holeboard task and T-maze. Overall, chickens breed specifically for low mortality displayed lower levels of fearfulness than the control individuals. McBride et al. (52) found that Welsh mountain sheep spent more time looking and touching their self-image in a mirror than two other breeds of sheep. The authors suggest this may have been due to breed differences in exploration and social tendencies (52). Veissier et al. (68) found breed differences in an observational learning task in female cattle, with more Limousin heifers learning the task than Aubrac heifers in the same experiment. The difference in task success between the two groups appeared to be due to differences in fearfulness between the breeds, with Aubrac heifers spending more time trying to escape the experimental room, rather than engaging with the task. Kendrick et al. (57) found Dalesbred and Clun forest sheep differed in their performance on a vocal discrimination task. In this case, the authors suggested that this could be due to the differing habitats of the breeds. Hill sheep have better abilities in vocal discrimination tasks, possibly due to being more dispersed in their natural habitat than lowland sheep, thus requiring more reliance on the use of vocalization to discriminate individuals when widely dispersed.

Many studies investigating cognitive performance standardize for breed differences by using just a single breed. As such, there are relatively few studies that directly compare across breeds. Perhaps also due to publication bias, it is possible that such studies have been conducted but no significant results found, leaving few published studies with an absence of breed differences to draw upon as examples. One example of such a lack of difference is in Murphy et al. (140), who compared Göttingen miniature pigs and standard commercial breed pigs in a judgment bias task and found no difference in their abilities to discriminate between auditory cues associated with positive and negative outcomes.

At this time, it is not possible to draw firm conclusions about the role of breed in cognitive performance, simply because there have been so few studies published where this has been directly compared between breeds. However, drawing from the studies that have found a breed-related association with performance, the results suggest that cognition is influenced by an animal's evolutionary, ecological and developmental environment (102).

### Personality

Animal personality is defined as moderately consistent individual differences in behavior across time and contexts (98, 141). A number of terms have been used to capture individuals' consistent patterns of behavioral, physiological and/or neuroendocrine profiles, including coping styles and behavioral syndromes. Personality research has traditionally focused on traits such as exploration, boldness, activity levels, sociability and aggressiveness (142). While moderately stable across time and contexts, personality also interacts with behavioral plasticity and predictability [i.e., within-individual change (143)].

Two common categorizations of personality types or coping styles are *proactive* and *reactive*. Proactive individuals are bolder and more exploratory than reactive individuals, allowing them to learn quickly in new situations but become relatively inflexible when previously learned rules change (144). By contrast, reactive individuals demonstrate greater behavioral flexibility than proactive individuals. A predominant hypothesis about the relationship between personality and cognition is that proactive individuals prioritize speed over accuracy in decision making (145). For instance, Nawroth et al. (79) report that goats scoring higher for exploration and sociability (consistent with proactive personality types) performed worse in tasks of object permanence and visual discrimination. Reactive laying hens also learned to associate a color-cue with a reward better than proactive hens (146). In fish, White et al. (27) found a negative correlation between boldness and learning to use cues to find hidden food in brook trout. Bensky et al. (147) report that bolder three-spined sticklebacks were quicker to learn a color discrimination task than shyer individuals, although no evidence was found for shyer individuals to perform better when the task was altered.

Griffin et al. (97) note that discerning robust relationships between an individual's personality and cognitive style will require tests of both cognitive abilities and personality traits to allow the full array of competing alternative hypotheses to be tested. This may lead to a multi-method multi-trait approach across both personality and cognition tests (148) to ensure the validity and robustness of relationships between personality and cognitive measurements.

### Mood

Affective state and cognition are deeply intertwined, with cognition influencing affective state and affective state in turn influencing cognitive processes (149). Affective state can be categorized into emotion and mood. Emotions are short-lived mental states that arise in response to rewarding or punishing stimuli (150). Emotions change rapidly and contribute to within individual variability in test performance (151). Moods, on the other hand, are longer-term mental states that are not tied to a specific stimulus and are thought to be the result of the accumulation of affective experiences in the mid- to longer-term past (152, 153). Moods are more specific to the individual and may contribute to between individual variability on cognitive tasks. Mood can also interact with personality to affect cognitive processes. For example, more reactive individuals in a negative mood judge novel information more negatively than reactive individuals in a positive mood, whilst proactive individuals' mood did not affect their judgements (154).

Mood affects information processing by altering response thresholds to stimuli (152). This has most commonly been evaluated in situations of ambiguity and is linked to risk taking. In particular, the cognitive bias test has been widely applied with farm animals to investigate the effect of mood on decision making under ambiguity [for a review see Baciadonna and McElligott (155)]. These tests have been devised to assess emotional state rather than cognition, and thus they cannot directly answer questions on the effect of mood on cognition. In addition, the question of how emotion may affect other cognitive processes, such as social learning, spatial cognition, or working memory, has not been assessed in farm animals to our knowledge. Affective states influence a wide range of cognitive processes in humans, such as self-regulation, information processing and decision making [e.g., see Martin and Clore (156)]. This suggests that mood may also be a source of within-individual variation in performance in animals, in tests measuring cognitive abilities other than those involved in risk taking. However, it is difficult to disentangle the effects of the specific test set-up from the effects of mood alone. Such as in tests of visual discrimination of faces, an animal's ability may be affected by how aversive they find the stimulus, which in turn impacts their ability to attend to the stimulus for long enough to complete the task. For example, horses spent less time looking at agonistic conspecific faces than neutral or positive conspecific faces (157). Whilst Lee et al. (158) showed that less anxious sheep spent less time attending to a threat stimulus than anxious sheep. Thus, if mood affects how aversive a stimulus is to attend to, this could affect performance on cognitive tasks requiring a certain level of attention toward specific stimuli.

Both genetic predisposition and environmental factors cause variation in mood. Whilst the former can be partially controlled for by using individuals of the same breed and genetic line, controlling for the environment can be less reliable due to the stochastic nature of life. In addition, genetic × environment interactions can lead to further variability at the individual level (108). To complicate matters further, we do not completely understand how mood is generated, and thus cannot fully control for it in cognition studies. Eldar et al. (159) proposed the theory that mood is the cumulative result of differences in expectations and the obtained outcomes of recent experiences. Raoult et al. (151) did not find strong evidence for this theory in their review of 95 papers on cognitive bias and manipulations used to affect mood. However, further research is required with more precise and overt tests of the predictions from this theory, as the studies reviewed did not have the original aim of testing Eldar's theory. Future cognitive studies could also benefit from assessing mood alongside the specific cognition test in which they are interested, as this would provide valuable information on the contribution of differences in mood to the variability found in tests of cognitive abilities.

### Motivation

In order that an animal completes a cognitive task, it must be motivated to engage and perform. Levels of motivation can differ both within and between individuals as a result of multiple factors: the reward type and timing, protocols used to induce motivation, and the inherent value of completing the task, all of which may be influenced by previous experience.

If correct trials are to be reinforced, the first consideration is the researcher's choice of reward. In farmed species, common rewards include access to food or conspecifics. When using food, providing a reward distinct from that of an animal's standard feed may increase motivation for some individuals and decrease it for others, dependent on individual preferences. For some non-livestock animals, it has been shown that using a preferred reward can increase motivation (160–162) but that preferences change over time [e.g., orangutans: Clay et al. (163)]. Therefore, depending on the length of the testing period, variation in task performance may reflect changes in an individual's reward preference.

When using appetitive rewards, the levels of pre-task satiety can influence the animal's willingness to participate. To induce motivation, animals may be food restricted prior to testing, such that access to food becomes more appealing. Blanket protocols are often applied across a study group (e.g., restrict test subjects to 70% of *ad libitum* intake or provide a set volume of food). It cannot be expected that each animal will respond equally to a fixed restriction, resulting in variability in levels of motivation and thus in perceived cognitive ability. If restrictions are not staggered, the first animal to be tested may be less motivated than the final individual, given the difference in total restriction time. Similarly, in cases where restriction protocols are not applied, the time since last feeding may also impact any appetitively rewarded trials. A final consideration is fluctuations in motivation over the course of a testing session. If a session requires many iterations of a task, the reward value may depreciate and, subsequently, trials carried out at the beginning of testing may not be comparable to those performed at the end.

As an alternative to food rewards, some social species may be rewarded socially by providing access to conspecifics—for example fish (30) and sheep (20) in maze-based tasks. Introducing conspecifics may mitigate some of the confounding factors of using appetitive rewards, but social reinforcers bring complications of their own. For example, motivation to gain access to a conspecific may be partially affected by social rank, as has been shown in non-human primates (164). Levels of motivation may also be influenced by the degree of contact offered as a reinforcer; calves were more motivated to perform an operant conditioning task for full contact with a conspecific, than for only contact with the head (92). Given that livestock animals can have preferences for certain group mates or familiar animals [e.g., cows: (165); sheep: (166, 167)] the identity of the “reward animal” is also of importance. Rewarding with a preferred or non-preferred individual could alter the perceived outcomes of the task. Interestingly, social interactions can also impact on the motivation to work for food rewards. Pedersen et al. (168) demonstrated that isolating a pig from its pen mate decreased the value of a food reward, highlighting the impact of social context on cognitive trial outcomes.

The type of task and the animal's perception of it may also influence motivation. Some tasks may have an inherent motivational value, regardless of any reward received for completion of a correct trial. For example, de Jonge et al. (169) found evidence of contra-freeloading in domestic pigs, meaning that the pigs preferred to work for food despite identical food being freely available. However, such a task may be cognitively stimulating to one animal, but not to another. It is also possible that motivation to perform may depend on living conditions. If a task is novel or enriching, an individual from a complex, enriched home environment may not find the task as rewarding as would an individual from a less stimulating home environment.

In all of the above examples, the researcher risks measuring motivation to engage with the task, rather than judging the cognitive ability of an individual. This confound is perhaps most important to consider when using latency to give a correct response as a measure of cognition, e.g., in maze completion.

### Diet

Diet and access to food can influence cognitive function, for example feed restriction, which is known to impact on learning and some aspects of memory in rodents (170, 171). Feed restriction during gestation, even for a short period of time, can have lasting effects on behavior and cognition in offspring. A study by Erhard et al. (103) investigated the impact of temporary feed restriction during early gestation in Scottish Blackface sheep. For this study, a control group was compared with a treatment group whose mothers had their feed intake reduced by 50% for the first 95 days of gestation. Although there was no difference in the average birth weight of the control and the treatment groups, lambs that experienced prenatal feed restriction were more active than control animals in novel object, social isolation, physical restraint, and suddenness tests. This increase in emotional reactivity also affected performance in cognitive tasks with prenatal feed restricted individuals less likely to learn the first reversal task in the T-maze if they had high levels of locomotion during social isolation and novel object tests. Similarly, there is evidence that the early life diet of an individual can influence cognitive performance, which, as shown by some studies, has the potential to last into adulthood (39, 172). For example, Rytych et al. (173) found that severely iron deficient piglets could not acquire a spatial T-maze task. Similarly Antonides et al. (174) found that iron deficient piglets had reduced reference memory in a holeboard task in comparison to non-iron deficient piglets.

The nutritional content of feed and the time period of exposure can have a significant impact upon cognitive function. In the context of biomedical research, several cognition studies have been conducted using pigs to investigate the effect of the “Western”-style diet, comprising high energy, high fat, and high sugar levels. Both Val-Laillet et al. (175) and Clouard et al. (176) found that prenatal exposure to a “Western”-style diet improved both working and reference memory in piglets, in comparison to piglets on a standard diet. However, there was no effect on cognitive function in piglets fed this diet during the early postnatal period with no previous exposure during gestation (176). Although this diet is typically not applicable for livestock, it highlights the influence that nutrition can have. However, other dietary constituents in the mother's diet during gestation and lactation can impact upon offspring cognition. Examples of these include sialic acid which improved learning and memory (177, 178) whilst iron which was shown to impair reference memory in piglets (173).

Diet will inevitably vary between life stages, however it is worth noting that this can be a cause of cognitive variation, especially when comparing results between two studies of the same species. In addition, diet directly impacts the gut microbiome, which is also closely linked to the brain and cognitive function.

### Gut microbiome

The gut is inhabited by trillions of microbes (the microbiota) and the term “gut microbiome” refers to their genetic material and capabilities. Microbiomics is a rapidly developing field, as interest in the broader effects of diet continues to grow. For example, a search of the literature published over the last 10 years shows an increase from 16 papers in 2007 to 2,210 papers in 2017 using the search term “gut microbiome” (Web of Science). It is outside of the scope of this review to fully evaluate the links between the gut and brain, but some relevant ideas are discussed in this section.

The gut microbiome and the brain communicate bidirectionally via multiple suggested mechanisms, known collectively as the microbiome-gut-brain axis [see comprehensive reviews from Mayer (179) and Galland (180)]. These lines of communication substantiate the idea that the gut may influence cognitive function. Many studies of the microbiome-gut-brain axis and its relationship to cognition have focused on the context of aging, disease and/or neurodisability. For example, some researchers use cognitive function to measure the efficacy of an intervention or as an indicator of the neurological impairments associated with, for example, Alzheimer's (181), diabetes (182), and autism (183). Although these studies are not directly comparable to livestock, they give an indication of how differences in the gut microbiome, caused by illness or physiological disruption, may lead to variability in cognitive function.

In addition to disease syndromes, disruption of the gut microbiome is linked with stress [e.g., Bailey et al. (184); O'Mahony et al. (185); Jašarević et al. (186)], and stress has known impacts on cognition (187). It may therefore be considered that an animal suffering stress could perform poorly in a cognitive task, either as a direct neurological consequence of the stress, or via changes in the composition of the gut microbiota. Indeed, Weinstock (188) showed that male mice exposed to prenatal stress showed signs of cognitive deficits—this was attributed in part to the mother's vaginal microbiome, which in turn influences the offspring's gut microbiome (186, 189).

The complex relationships between gut health, diet, stress, illness, and cognition are further complicated by the fact that the gut microbiome does not remain stable throughout life and can be influenced by a variety of factors including birth conditions, diet, environment, disease and aging (190–192). These multi-level interactions make it difficult to suggest that variability in livestock cognitive performance could be attributed to the microbiome, but it is nevertheless something important to consider as part of a wider system.

## Accounting for individual variation

Accounting for individual variation in cognition is important in farmed animal species, and applied ethology in general, because we are often interested in how individuals experience their environment, not just a population. Aggregating and analysing data across individuals can lead to misleading conclusions when the goal is to understand individual-level processes. For example, not accounting for within-individual variation in cognitive change risks committing an ecological fallacy (193). That is, incorrectly inferring the form of within-individual processes (e.g., the relationship between stress and cognitive ability) from results pertaining to group-level, aggregated patterns of change. Extreme cases may lead to Simpson's paradox, where the relationships between variables at an aggregate level are the reverse of those relationships at lower levels of scale. In animal welfare science, evidence suggests that most behavioral variation is explained by individual variation rather than by higher-level factors such as groups or pens [e.g., chickens: (194)], so appropriately incorporating individual variation is key.

Animal cognition studies often record repeated measurements on individuals, which are then used to quantify summary measures of cognitive performance, such as the number of trials needed to learn a task or the average probability of responding correctly. However, this often precludes estimating between- and within-individual variation as a result of data aggregation across repeated measurements. In addition, when the goal is to quantify the relationship between individual variation in cognition, and endogenous and exogenous factors (such as those discussed above), researchers may be motivated to conduct a number of separate statistical analyses. Yet, conducting multiple analyses on the same data set can lead to increased chances of false positives. This is further complicated by the low sample size of animal cognition studies (195), which are not only at increased risk of Type II errors (i.e., not enough signal to reject the null hypothesis), but also Type I errors (i.e., incorrectly rejecting the null hypothesis), and errors of sign and magnitude (196). For example, a significant *p*-value in a small sample size study should not be taken as evidence of a robust effect (197).

Accounting for individual variation may further improve the reproducibility of studies. In recent years, the reproducibility of scientific findings has received increasing scrutiny, most notably in the psychological sciences (197) but also in a number of other areas [e.g., cancer biology; (198); economics: (199); artificial intelligence: (200)]. Conditions for irreproducibility include studies with low sample sizes and small true effect sizes for the relationships being investigated, along with questionable research practices such as data dredging or p-hacking (running analyses multiple times until a significant *p*-value is found) and poor research incentives (201). Studies of farmed animal cognition may also be at risk of irreproducibility due the small number of animals used, especially when potentially large systematic individual variation in cognitive performance is not accounted for, making across-study results inconsistent. Indeed, Voelkl and Wurbel (202) argue that a key condition for irreproducibility in pharmacological studies could be the lack of appropriate estimates of phenotypic variation, and argue that greater attention should be paid to quantifying phenotypic reaction norms.

In this section, we highlight how multilevel models can be used to investigate individual variation across repeated measurements. While multilevel models are neither the only approach for measuring variation nor particularly new (203), their use is increasingly encouraged as the state-of-the-art approach in accounting for variation across distinct clusters (e.g., individuals) in a range of disciplines [e.g., animal welfare: (204); ecology: (205); human evolutionary ecology: (206); psychology: (207); health: (208)]. This includes analysing individual variation in behavior (e.g., personality) and reaction norms in behavioral ecology (209), and individual variation in human cognition (210). Adopting multilevel models for assessing individual variation in cognition in farmed animals is a natural extension. Below, and in the Supplementary Materials (available on Github: https://github.com/ConorGoold/Bushby-et-al-individual-variation-cognition), we demonstrate how multilevel models may be applied to explore facets of individual variation in animal cognition.

### Multilevel models

Multilevel models extend the general linear model framework to account for variation across different groups or clusters, such as repeated measurements on individuals. Specifically, deviations for each clustering unit are estimated from the population-level intercept, slope and/or residual standard deviation parameters (known as “random effects”). These deviations are constrained by their own (usually normal) distribution, which improves the predictive ability of these models compared to non-multilevel models through the effects of *partial-pooling* (203). The deviations represent the amount of individual variation, which in turn can be predicted by a number of “cluster-level” predictor variables. For example, in studies of behavior, variation among individuals in the intercept parameter is used to operationally define animal personality, variation among individuals in the slope parameter across an environmental gradient defines behavioral plasticity, and variation among individuals in the residual variation captures behavioral predictability. Together, the analysis of these sources of variation is referred to *behavioral reaction norms* (211, 212). Variation in these parameters can, in turn, be predicted by individual-level predictor variables such as sex, age, or life stage. Behavioral repeatability is calculated using the intraclass correlation coefficient: the random intercept variance divided by the total model variance (213).

As an example case, imagine a reversal learning task, where we first teach individuals an initial contingency and then reverse this contingency to assess cognitive flexibility. The data are a series of binary (0/1 values, i.e., Bernoulli distributed) trials for each individual indicating whether they completed the task on each trial correctly or incorrectly. We may also be interested in whether individual variation is affected by some independent variable, such as personality type (e.g., reactive or proactive), diet (e.g., Western vs. non-Western diet) or sex. To compare the different groups, one option is to summarize the data for each individual by the difference in the number of trials taken to learn the initial and reversal contingencies, and estimate the relationship between this summary measure and group (e.g., using an independent samples *t*-test). However, this analysis has a number of drawbacks. Firstly, it cannot distinguish between- from within-individual variation. Secondly, it splits the data analysis into multiple stages that may limit reproducibility (Gelman and Loken, unpublished manuscript). Thirdly, it requires defining a potentially arbitrary criterion to decide whether the task was learned by each individual, which could lead to throwing out data for those individuals not matching that criterion. For instance, only 29 out of 64 sheep in Erhard et al. (103) met the required learning criterion in a reversal learning T-maze task, meaning subsequent analyses were conducted on varying numbers of individuals while other data was dropped from the analysis. van Horik et al. (214) also discuss selection biases in participation rates of cognitive tests in pheasant chicks, which were dependent on sex, personality and body condition.

**Figure 1:**
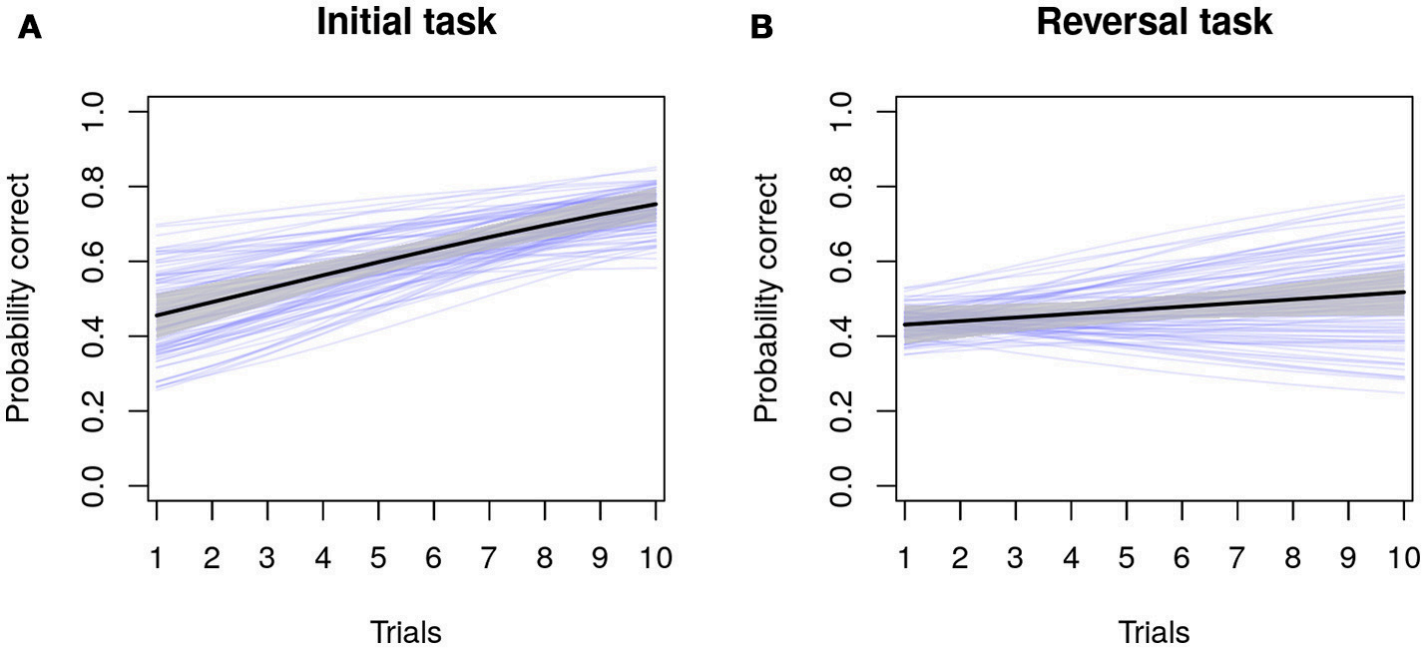
Model results from a hypothetical study of individual variation in cognition. Dark black lines and gray areas show the population-average probability and its 89% credible interval of choosing a correct answer across trials in an initial learning task **(A)**, and reversal learning task **(B)**. Blue lines show estimates for each individual (*n* = 100), for which variance parameters and individual predictions can be directly compared.

Figure 1 presents the results of a (Bayesian) multilevel logistic or Bernoulli regression model for our hypothetical example study. For those unaccustomed with fitting multilevel models, a formal description of this analysis is presented in the Supplementary Materials (available on Github: https://github.com/ConorGoold/Bushby-et-al-individual-variation-cognition), including an R script file for simulating the data, running the analyses (both Bayesian and frequentist approaches), and producing the figures. In Figures 1A,B, the black lines indicate the population average change in the probability of a correct choice, the gray shaded region illustrates the 89% Bayesian credible interval around the population average (i.e., the 89% most likely parameter values), and the thinner blue lines demonstrate the individual-level regression lines (the posterior means) for each individual (*n* = 100). Figure 1A demonstrates the probability correct across trials in the initial task and (Figure 1B) the probability correct across trials in the reversal task, with the average probability and the rate of learning being lower in the reversal task across individuals. As can be seen from the dispersion of the blue regression lines, there is individual variation in the parameters (both average probabilities and the rate of learning across trials). In the reversal learning task, some individuals' probabilities of responding correctly become worse across trials, despite the population-average slope being positive. From this model, the variance of the different random effect parameters can be extracted and compared directly using the Bayesian posterior distribution. Coefficients describing the linear relationship between individual-level predictor variables and individual variation in learning rates (see the Supplementary Material for further examples on Github: https://github.com/ConorGoold/Bushby-et-al-individual-variation-cognition), can also be investigated.

Multilevel models are flexible tools for a range of data types, including unbalanced designs and more complicated cases with multiple dependent variables, such as multilevel structural equation models or multilevel network models [e.g., (215)]. Practically, it is recommended to have at least 100 individuals to accurately estimate individual variation (216), although estimates of cluster-level variation in Bayesian multilevel models tend to be more accurate in small sample data sets than frequentist models using maximum likelihood estimation. In addition, an advantage of Bayesian estimation is that we can estimate the uncertainty (via Bayesian credible intervals) in cluster-level parameters (e.g., individual-level predictions), meaning estimates from smaller sample sizes may just be more uncertain rather than inaccurate. Fortunately, as demonstrated in the Supplementary Material (available on Github: https://github.com/ConorGoold/Bushby-et-al-individual-variation-cognition), fitting Bayesian models is becoming just as easy as frequentist models in common statistical software.

Finally, statistically accounting for individual variation is just one component needed to ensure reproducibility of scientific findings. Due to ethical and practical limitations, simply obtaining larger sample sizes in farmed animal cognition studies may not be realistic. Instead, researchers should consider pre-registering their studies to limit questionable (but often unconscious) research practices such as data dredging. Moreover, replication experiments and cross-lab collaboration efforts could help to confirm key hypotheses in the field [e.g., (217)] and make use of a larger number of subjects without increasing the sample size per study unnecessarily.

## Conclusion

We have reviewed factors causing within- and between-individual variation in cognitive testing of farmed animal species and demonstrated how to account for individual variation using multilevel models. We emphasize the importance of taking into consideration other factors that could cause variation and the importance for accounting for individual variation to ensure the reproducibility of farm animal cognition and cognition studies in general.

## Author contributions

EB, MF, CG, HG, LS, and LC contributed to writing and editing this manuscript. Supplementary material was provided by CG.

### Conflict of interest statement

The authors declare that the research was conducted in the absence of any commercial or financial relationships that could be construed as a potential conflict of interest. The reviewer MZ and handling Editor declared their shared affiliation.
